# Supplementary material for: Lacticaseibacillus rhamnosus Fermentation Ameliorates Physicochemical Properties, Physiological Activity, and Volatile and Non-Volatile Compounds of Mango Juice: Preliminary Results at Laboratory Scale
Source: Foods. 2025 Feb 12;14(4):609. doi: 10.3390/foods14040609 (PMC11854518; doi:10.3390/foods14040609)

Supplementary data

Figure S1 Principal component analysis of correlation loadings plots based on the data of volatile compounds detected in the unfermented and fermented mango juice.

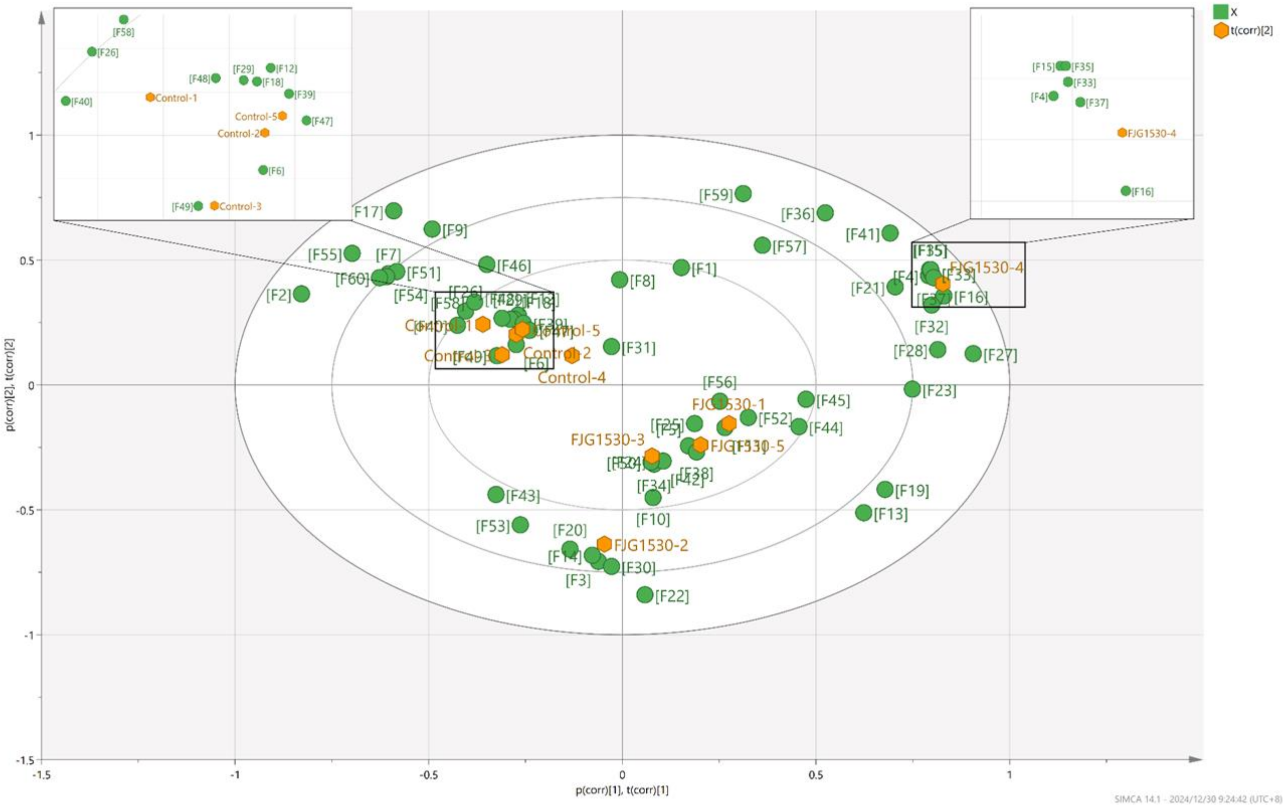

Figure S2 volcano plot of differential metabolites in mango juice between the unfermented and fermented mango juice. The ESI+ model (A); the ESI- model (B).

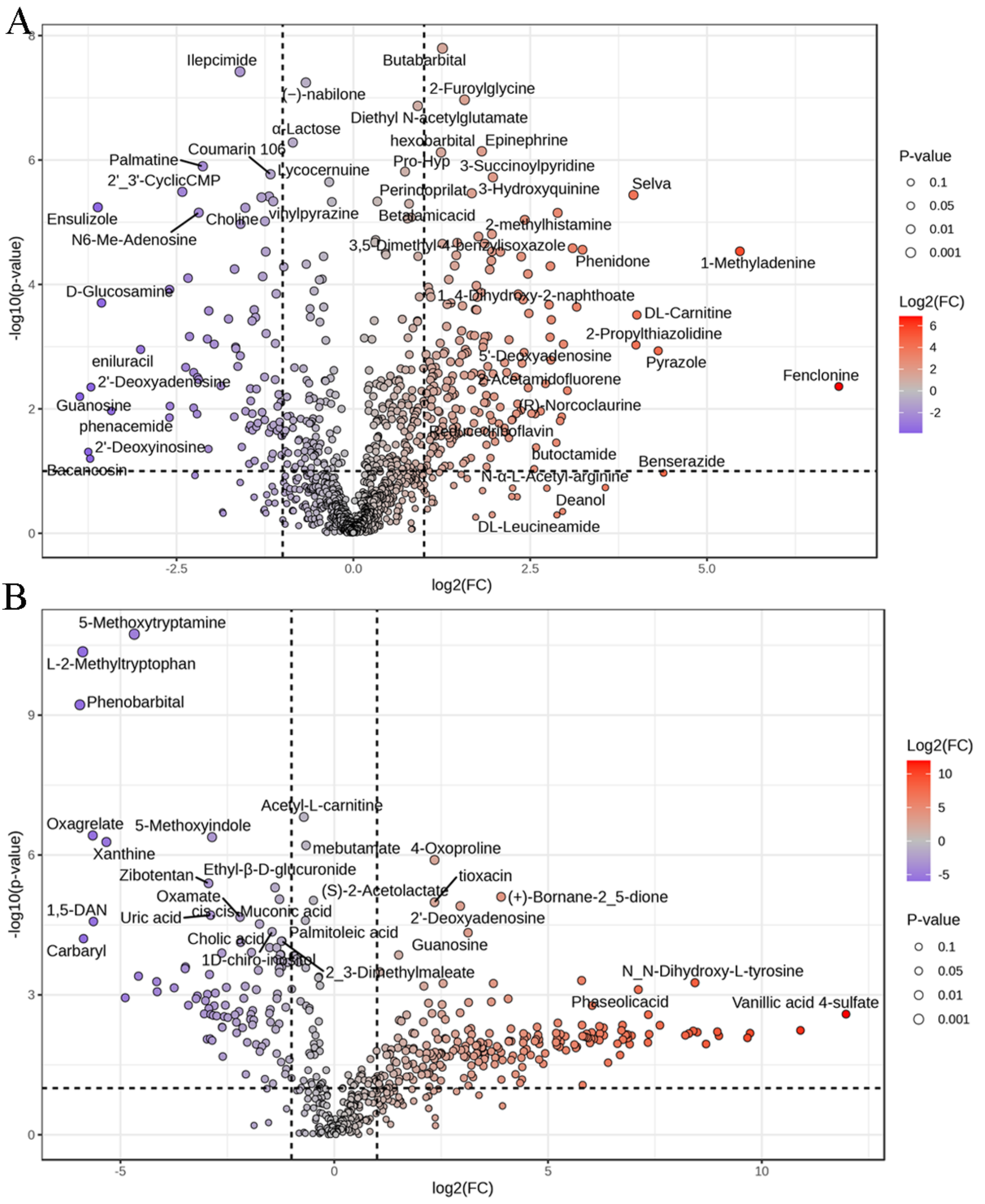

Supplement: Supplementary file 1 [file foods-14-00609-s001.zip › foods-3448058-supplementary.pdf]
